# Supplementary material for: In silico biophysics and rheology of blood and red blood cells in Gaucher Disease
Source: PLoS Comput Biol. 2025 Sep 10;21(9):e1012705. doi: 10.1371/journal.pcbi.1012705 (PMC12435781; doi:10.1371/journal.pcbi.1012705)
Supplement: S1 Text — (PDF) [file pcbi.1012705.s001.pdf]

In silico biophysics and rheology of blood and red blood cells in Gaucher Disease

Zhaojie Chai, Guansheng Li, Papa Alioune Ndour, Philippe Connes, Pierre A. Buffet, Melanie Franco, George Em Karniadakis

S1_Text. Dissipative particle dynamics (DPD) method and particle-based blood cell models

In this study, we use the dissipative particle dynamics (DPD) approach to simulate blood flow, specifically focusing on plasma and red blood cells (RBCs). The DPD technique operates at a mesoscopic scale and is a particle-based simulation method where each DPD particle represents a group of molecules. These particles interact through soft pairwise forces that rely solely on their relative positions and velocities [1, 2]. As a result, DPD accurately captures the hydrodynamic behavior of fluids at this scale and has proven effective in studying complex fluids [3, 4]. Each particle i follows motion equations governed by pairwise interactions, f_i, with nearby particles j, and the dynamics are integrated using a velocity-Verlet algorithm. Newton's second law dictates the time evolution of each particle's velocity (v_i) and position (r_i), based on its mass (m_i):

$$d\mathbf{r}_i = \mathbf{v}_i\, dt, \quad d\mathbf{v}_i = \frac{\mathbf{f}_i}{m_i}\, dt. \tag{1}$$

In the standard DPD approach, the total force $\mathbf{f}i$ acting on particle i due to particle j consists of three components: a conservative force ($\mathbf{F}^C ij$), a dissipative force ($\mathbf{F}^D_{ij}$), and a stochastic or random force ($\mathbf{F}^R_{ij}$).

$$\mathbf{F}^C_{ij} = a_{ij}\left(1 - \frac{r_{ij}}{r_c}\right)\hat{r}_{ij} \quad \text{for } r_{ij} \le r_c; \quad \mathbf{F}^C_{ij} = 0 \quad \text{for } r_{ij} > r_c, \tag{2}$$

$$\mathbf{F}^D_{ij} = \gamma\, \omega_d(r_{ij})(\hat{r}_{ij} \cdot \hat{v}_{ij})\,\hat{r}_{ij}, \tag{3}$$

$$\mathbf{F}^R_{ij} = \sigma\, \omega_r(r_{ij})\, \zeta_{ij} \sqrt{dt}\, \hat{r}_{ij}, \tag{4}$$

A cut-off radius, denoted r_c, is applied in the model, where the terms a_{ij}, γ, and σ serve as the coefficients for the conservative, dissipative, and random interactions, respectively. The distance between particles is represented by r_{ij} along with its corresponding unit vector $\hat{r}_{ij}$, while $\hat{v}_{ij}$ captures the relative velocity between particles. The term ζ_{ij} represents a Gaussian random variable with zero mean and unit variance, and dt is the time increment for the simulation. The fluctuation-dissipation theorem links the parameters γ and σ along with the weighting functions, adhering to the relationships $\omega_d = \omega_r^2$ and $\sigma^2 = 2\gamma k_B T$, where k_B is the Boltzmann constant and T signifies the system's temperature. The weighting function, $\omega_r(r_{ij}) = \left(1 - \frac{r_{ij}}{r_c}\right)^k$, has $k = 1$ in standard DPD, though other k values may be used to alter fluid viscosity [5].

Table S1 provides details on the DPD parameters chosen for the fluid particles in this study. For an in-depth exploration of the DPD technique, refer to [2, 6].

Table 1. DPD fluid parameters used in the current study: n is the fluid's number density, a_C is the conservative force coefficient, γ is the dissipative force coefficient, and k is the weight function exponent. In all simulations, we set the particle mass $m = 1$, and the thermal energy $k_B T = 0.1$ in DPD units.

n	r_c	a_C	γ	k
4	2.0	5.0	30.0	0.2

In addition to blood plasma, which is represented as a collection of free DPD particles, the red blood cell (RBC) membrane is modeled using a two-dimensional triangulated network comprising N_v vertices (DPD particles). These vertices are linked by N_s elastic bonds, which enforce the appropriate mechanical properties of the membrane. This DPD-based approach to representing RBCs has been extensively validated in prior studies for both normal and pathological cells [3, 7]. The free energy of a single cell, V_{cell}, is expressed as

$$V_{\text{cell}} = V_s + V_b + V_{a+v}. \tag{5}$$

The elastic energy V_s, which characterizes the elastic interactions within the cell membrane, is defined by

$$V_s = \sum_{j \in 1 \dots N_s} \left[\frac{k_B T l_m (3x_j^2 - 2x_j^3)}{4p(1 - x_j)} + \frac{k_p}{l_j} \right], \tag{6}$$

where p represents the persistence length, k_p denotes the spring constant, $k_B T$ is the unit of thermal energy, l_j is the length of the j-th spring, l_m represents the maximum extension of the spring, and $x_j = \frac{l_j}{l_m}$. The values of p and k_p are determined by balancing equilibrium forces and by their relationship with the macroscopic shear modulus, E_s:

$$E_s = \frac{\sqrt{3} k_B T}{4 p l_m x_0} \left(\frac{x_0}{2(1 - x_0)^3} - \frac{1}{4(1 - x_0)^2} + \frac{1}{4} \right) + \frac{3\sqrt{3} k_p}{4 l_0^3}, \tag{7}$$

where l_0 denotes the equilibrium length of the spring, and $x_0 = \frac{l_0}{l_m}$. The bending resistance V_b of the cell membrane is modeled by

$$V_b = \sum_{j \in 1 \dots N_s} E_b [1 - \cos(\theta_j - \theta_0)], \tag{8}$$

where E_b is the bending modulus, which is related to the macroscopic bending rigidity k_c through the expression $E_b = \frac{2k_c}{\sqrt{3}}$. Here, θ_j represents the instantaneous angle between two adjacent triangles sharing a common edge j, and θ_0 denotes the spontaneous angle. Additionally, area and volume constraints, V_{a+v}, are applied to simulate the area-conserving lipid bilayer and the incompressible internal fluid. The associated energy is expressed as

$$V_{a+v} = \sum_{j \in 1 \dots N_t} \frac{k_d (A_j - A_0)^2}{2A_0} + \frac{k_a (A_{\text{cell}} - A_{\text{tot}}^0)^2}{2A_{\text{tot}}^0} + \frac{k_v (V_{\text{cell}} - V_{\text{tot}}^0)^2}{2V_{\text{tot}}^0}, \tag{9}$$

where N_t represents the number of triangles in the membrane network, A_0 is the equilibrium area of a triangle, and k_d, k_a, and k_v are the coefficients for local area, global area, and volume constraints, respectively. The terms A_{tot}^0 and V_{tot}^0 denote the target values for cell area and volume.

In this study, we model a typical RBC with $N_v = 500$. The chosen cell surface area and volume for a normal RBC are $A_{\text{tot}}^0 = 132.9\,\mu\text{m}^2$ and $V_{\text{tot}}^0 = 92.5\,\mu\text{m}^3$, resulting in a

surface-to-volume ratio $S/V = 1.44$. A complete list of parameters used in our RBC model is provided in Table S2, and these values have been calibrated against experimental data and validated through simulations covering both single RBC mechanics and blood flow dynamics [3, 8, 9].

The DPD parameters applied in equations (2)-(4) for all DPD particle types, along with the membrane parameters in equations (6)-(9) for different blood cell models, are listed in Tables S2 and S3, respectively.

In addition, to prevent RBC membranes from overlapping, we applied a repulsive term of Lennard-Jones potential to all membrane vertices [8]; this potential is given by

$$U(r) = \begin{cases} 4E\left[\left(\frac{\sigma}{r}\right)^{12} - \left(\frac{\sigma}{r}\right)^{6}\right], & r \le 2^{1/6}\sigma, \\ 0, & r > 2^{1/6}\sigma, \end{cases} \tag{10}$$

where E and σ are scaling constants for energy and distance, respectively, and these interactions vanish for $r > 2^{1/6}\sigma$.

Table 2. Cell membrane parameters for normal RBCs (CTR-RBC), and Gaucher Disease RBCs (GD-RBC) are defined as follows. N_v represents the number of DPD particles on the membrane, l_m is the maximum bond extension, l_0 is the equilibrium bond length, E_b^M denotes the bending modulus in our simulation, E_s^M stands for the shear modulus in our simulation, and A^0_{tot} and V^0_{tot} are the specified cell area and volume, respectively. The parameter $k_d + k_a$ represents the combined area constraint coefficient, while k_v is the volume constraint coefficient.

Cell	N_v	l_m/l_0	E_b^M	E_s^M	A^0_{tot} (V^0_{tot})	$k_d + k_a$ (k_v)
N-RBC	500	1.8	6.025	100.0	132.87 (92.45)	5000 (5000)
GD-RBC1	500	1.8	6.025	900.0	132.87 (92.45)	5000 (5000)
GD-RBC2	500	1.8	6.025	2000.0	132.87 (92.45)	5000 (5000)
GD-RBC3	500	1.8	6.025	400.0	132.87 (92.45)	5000 (5000)

Table 3. Parameters for interactions between different types of DPD particles are as follows. r_c represents the cut-off radius, a_{ij} is the conservative interaction coefficient, γ denotes the dissipative coefficient, and k is the exponent in the weight function. Note that S refers to the solvent (modeling plasma), and R represents CTR-RBCs, and GD-RBCs.

Type	r_c	a_{ij}	γ	k
S-S	2.0	5.0	30.0	0.20
S-R	1.5	0.0	45.0	0.20
R-R	1.0	10.0	10.0	0.20

In order to relate the DPD parameters to physical values, we first define the length and time scales. The RBC membrane shear modulus imposes the time scale for the DPD system, which follows

$$[t] = [L] \times \frac{\eta^P}{\eta^M} \times \frac{E_s^M}{E_s^P}, \tag{11}$$

where E_s is the RBC membrane shear modulus, η is the plasma viscosity, and superscripts M and P denote the model (DPD) and physical units, respectively. The length scale is taken as $[L] = 1 \times 10^{-6}$ m, whereas the time scale is evaluated using Eq. (11) as $[t] = 1.84 \times 10^{-3}$ s, based on the membrane shear modulus of healthy RBCs $E_s^P = E_s = 4.73 \times 10^{-6}$ N/m and plasma viscosity $\eta^P = 1.2 \times 10^{-3}$ Pa · s. The detailed parameter is consistent with the methodology of our previous studies [3].

References

1. Espanol P, Warren P. Statistical mechanics of dissipative particle dynamics. EPL (Europhysics Letters). 1995;30(4):191.
2. Espanol P, Warren P. Statistical mechanics of dissipative particle dynamics. Europhys Lett. 1995;30(4):191.
3. Fedosov DA, Pan W, Caswell B, Gompper G, Karniadakis GE. Predicting human blood viscosity in silico. Proceedings of the National Academy of Sciences. 2011;108(29):11772–11777.
4. Ye T, Phan-Thien N, Lim CT. Particle-based simulations of red blood cells—A review. Journal of biomechanics. 2016;49(11):2255–2266.
5. Fan X, Phan-Thien N, Chen S, Wu X, Yong Ng T. Simulating flow of DNA suspension using dissipative particle dynamics. Physics of Fluids. 2006;18(6).
6. Groot RD, Warren PB. Dissipative particle dynamics: Bridging the gap between atomistic and mesoscopic simulation. The Journal of Chemical Physics. 1997;107(11):4423–4435.
7. Cai S, Li H, Zheng F, Kong F, Dao M, Karniadakis GE, et al. Artificial intelligence velocimetry and microaneurysm-on-a-chip for three-dimensional analysis of blood flow in physiology and disease. Proceedings of the National Academy of Sciences. 2021;118(13):e2100697118.
8. Fedosov DA, Caswell B, Karniadakis GE. A multiscale red blood cell model with accurate mechanics, rheology, and dynamics. Biophysical journal. 2010;98(10):2215–2225.
9. Deng Y, Papageorgiou DP, Li X, Perakakis N, Mantzoros CS, Dao M, et al. Quantifying fibrinogen-dependent aggregation of red blood cells in type 2 diabetes mellitus. Biophysical journal. 2020;119(5):900–912.
