# Supplementary material for: In silico biophysics and rheology of blood and red blood cells in Gaucher Disease
Source: PLoS Comput Biol. 2025 Sep 10;21(9):e1012705. doi: 10.1371/journal.pcbi.1012705 (PMC12435781; doi:10.1371/journal.pcbi.1012705)
Supplement: S2 Text — (PDF) [file pcbi.1012705.s002.pdf]

In silico biophysics and rheology of blood and red blood cells in Gaucher Disease

Zhaojie Chai, Guansheng Li, Papa Alioune Ndour, Philippe Connes, Pierre A. Buffet, Melanie Franco, George Em Karniadakis

S2_Text. Cell-cell interaction potentials and measurements of membrane stiffness

Cell-cell interaction potentials

Consistent with our previous studies [1,2], the detailed parameter values for the Morse and Lennard-Jones (LJ) potentials are provided in Table 1.

Table 1. Model parameters for Morse potential and Lennard-Jones (LJ) potential.

Parameter	Simulation	Physical
r_0	1.0	1 μm
D_e	5.0	-
β	2.0	2.0 (dimensionless scaling factor)
σ	0.3	0.3 μm
ε	0.4	3.392×10^{-25} J
r_{LJ}	0.25	0.25 μm

Measurements of membrane stiffness

To quantitatively characterize the mechanical properties of our model, the stiffness of the lipid membrane is measured. In our simulations, we followed an approach analogous to experimental setups [3], simulating the adhesion of a single GD RBC to laminin $\alpha 5$ by initially attaching an RBC to a substrate. After allowing adequate contact time for the cell-surface interaction to stabilize, a pressure gradient was introduced to the blood plasma, creating shear flow along the X-axis, from left to right.

Subsequently, a stepwise increase in the pressure gradient was applied to the blood plasma until the X-axis length of the CTR RBC in the simulation aligned with that observed experimentally for the CTR RBC, measuring 11.6 μm. At this stage, the simulated RBC and the experimental RBC exhibited a comparable morphology. The pressure gradient in this simulation was found to correspond to a shear stress of 1 dyn/cm^2.

Once this shear stress was established, it was held constant at 1 dyn/cm^2 while varying the RBC's shear modulus in the simulations, ranging from $1Es_0$ to $25Es_0$. After each simulation, the X-axis length of the GD RBC was compared to the X-axis length of the GD RBC observed experimentally (Fig S1).

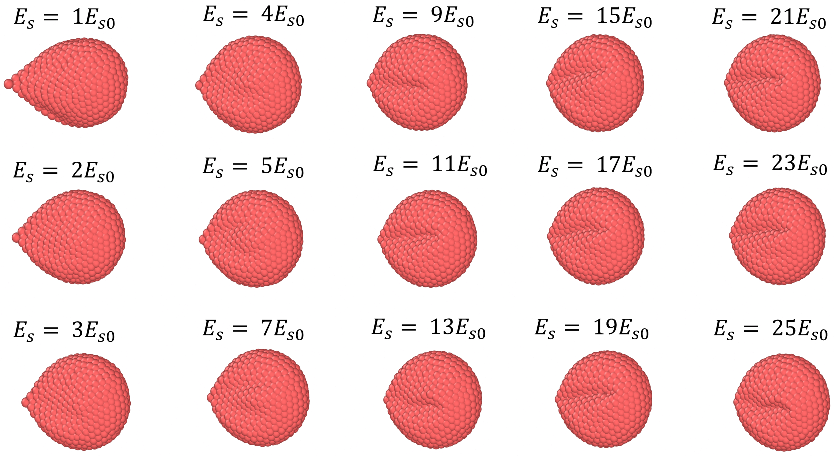

Fig S1. Morphology of RBC in simulations under different shear modulus conditions at a shear stress of 1 dyn/cm^2.

References

1. Deng Y, Papageorgiou DP, Li X, Perakakis N, Mantzoros CS, Dao M, et al. Quantifying fibrinogen-dependent aggregation of red blood cells in type 2 diabetes mellitus. Biophysical journal. 2020;119(5):900–912.

2. Chang HY, Yazdani A, Li X, Douglas KA, Mantzoros CS, Karniadakis GE. Quantifying platelet margination in diabetic blood flow. Biophysical journal. 2018;115(7):1371–1382.

3. Franco M, Collec E, Connes P, van den Akker E, Billette de Villemeur T, Belmatoug N, et al. Abnormal properties of red blood cells suggest a role in the pathophysiology of Gaucher disease. Blood, The Journal of the American Society of Hematology. 2013;121(3):546–555.
