# Supplementary material for: In silico biophysics and rheology of blood and red blood cells in Gaucher Disease
Source: PLoS Comput Biol. 2025 Sep 10;21(9):e1012705. doi: 10.1371/journal.pcbi.1012705 (PMC12435781; doi:10.1371/journal.pcbi.1012705)
Supplement: S3 Text — (PDF) [file pcbi.1012705.s003.pdf]

In silico biophysics and rheology of blood and red blood cells in Gaucher Disease

Zhaojie Chai, Guansheng Li, Papa Alioune Ndour, Philippe Connes, Pierre A. Buffet, Melanie Franco, George Em Karniadakis

S3_Text. Dimensionless Parameters

Dimensionless Parameters

To characterize the mechanical and flow regimes of our simulations, we define three key nondimensional parameters:

- **Capillary number** (Ca):
$$Ca = \frac{\eta \, \dot{\gamma} \, a_0}{E_s}, \tag{1}$$
where $\eta = 1.2 \times 10^{-3}\,\mathrm{Pa \cdot s}$ is the plasma viscosity, $\dot{\gamma}$ is the applied shear rate (ranging from 1 to $1000\,\mathrm{s}^{-1}$), $a_0 = 2.82 \times 10^{-6}\,\mathrm{m}$ is the characteristic RBC radius defined as the equivalent sphere radius based on RBC volume [1], and $E_s = 4.73 \times 10^{-6}\,\mathrm{N/m}$ is the membrane shear modulus of healthy RBCs.
- **Reynolds number** (Re) [2]:
$$Re = \frac{\rho \, \dot{\gamma} \, a^2}{\eta}, \tag{2}$$
where $\rho = 1000\,\mathrm{kg/m^3}$ is the fluid density and $a = 3.91 \times 10^{-6}\,\mathrm{m}$ is the RBC radius.
- **Nondimensional bending stiffness** (B^*), which characterizes the relative bending rigidity with respect to shear elasticity [3]:
$$B^* = \frac{E_b}{a_0^2 E_s}, \tag{3}$$
where $E_b = 2.4 \times 10^{-19}\,\mathrm{J}$ is the bending modulus.

In our simulations, for shear rates ranging from 1 to $1000\,\mathrm{s}^{-1}$, the dimensionless numbers for healthy RBCs span the following ranges: the Reynolds number Re ranges from 5.1×10^{-5} to 5.1×10^{-2}, the capillary number Ca ranges from 7.2×10^{-4} to 7.2×10^{-1}, and the nondimensional bending stiffness $B^* = 6.4 \times 10^{-3}$ remains constant.

These values confirm that the simulations are conducted in the low-Reynolds-number regime and cover a physiologically relevant range of mechanical stresses. The capillary number Ca quantifies the balance between viscous deformation and elastic resistance, indicating a transition from elasticity-dominated to viscosity-dominated behavior as shear rate increases. The low Re values validate the dominance of viscous forces. The small B^* reflects that membrane shear elasticity dominates over bending resistance in RBC deformation. These parameters collectively ensure that our simulation regime aligns with the mechanical environment of RBCs in microcirculation and support meaningful comparison with experimental and computational studies [1–4].

References

1. Rezghi A, Zhang J. Tank-treading dynamics of red blood cells in shear flow: On the membrane viscosity rheology. Biophysical journal. 2022;121(18):3393–3410.
2. Bagchi P, Johnson PC, Popel AS. Computational fluid dynamic simulation of aggregation of deformable cells in a shear flow. Journal of biomechanical engineering. 2005;127(7):1070–1080.
3. Yazdani A, Bagchi P. Influence of membrane viscosity on capsule dynamics in shear flow. Journal of fluid mechanics. 2013;718:569–595.
4. Fedosov DA, Pan W, Caswell B, Gompper G, Karniadakis GE. Predicting human blood viscosity in silico. Proceedings of the National Academy of Sciences. 2011;108(29):11772–11777.
