# Supplementary material for: In silico biophysics and rheology of blood and red blood cells in Gaucher Disease
Source: PLoS Comput Biol. 2025 Sep 10;21(9):e1012705. doi: 10.1371/journal.pcbi.1012705 (PMC12435781; doi:10.1371/journal.pcbi.1012705)
Supplement: S4 Text — (PDF) [file pcbi.1012705.s004.pdf]

In silico biophysics and rheology of blood and red blood cells in Gaucher Disease

Zhaojie Chai, Guansheng Li, Papa Alioune Ndour, Philippe Connes, Pierre A. Buffet, Melanie Franco, George Em Karniadakis

S4_Text. Effect of RBC Stiffness on Disaggregation Threshold

Effect of RBC Stiffness on Disaggregation Threshold

Our simulations show that the disaggregation threshold increases moderately with RBC shear modulus (Fig S2).. This suggests that while increased membrane stiffness can enhance the disaggregation threshold, it has a limited impact on the shear rate required to disrupt aggregates—consistent with prior experimental findings that reported minimal changes in critical shear stress despite significant increases in membrane rigidity [1, 2].

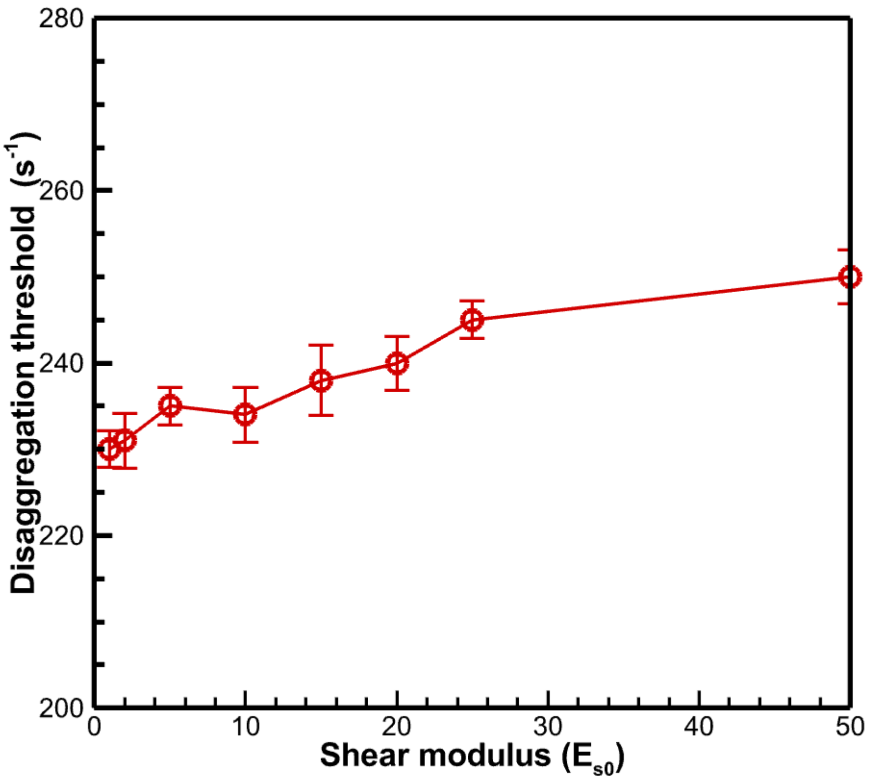

Fig S2. Influence of RBC stiffness on disaggregation threshold. The disaggregation threshold (in s^{-1}) is shown as a function of red blood cell (RBC) membrane shear modulus (E_s) normalized by the baseline value (E_{s0}). While a slight increasing trend is observed, the overall change remains modest. These results suggest that increasing RBC stiffness has a limited effect on the shear rate required to disrupt aggregates.

References

1. Maslianitsyna AI, Ermolinsky PB, Lugovtsov AE, Priezzhev AV. Study by optical techniques of the dependence of aggregation parameters of human red blood cells on their deformability. Journal of Biomedical Photonics & Engineering. 2020;6(2):020305.

2. Xue S, Lee BK, Shin S. Disaggregating shear stress: the roles of cell deformability and fibrinogen concentration. Clinical Hemorheology and Microcirculation. 2013;55(2):231–240.
