# Supplementary figures and images for: In silico biophysics and rheology of blood and red blood cells in Gaucher Disease
Source: PLoS Comput Biol. 2025 Sep 10;21(9):e1012705. doi: 10.1371/journal.pcbi.1012705 (PMC12435781; doi:10.1371/journal.pcbi.1012705)

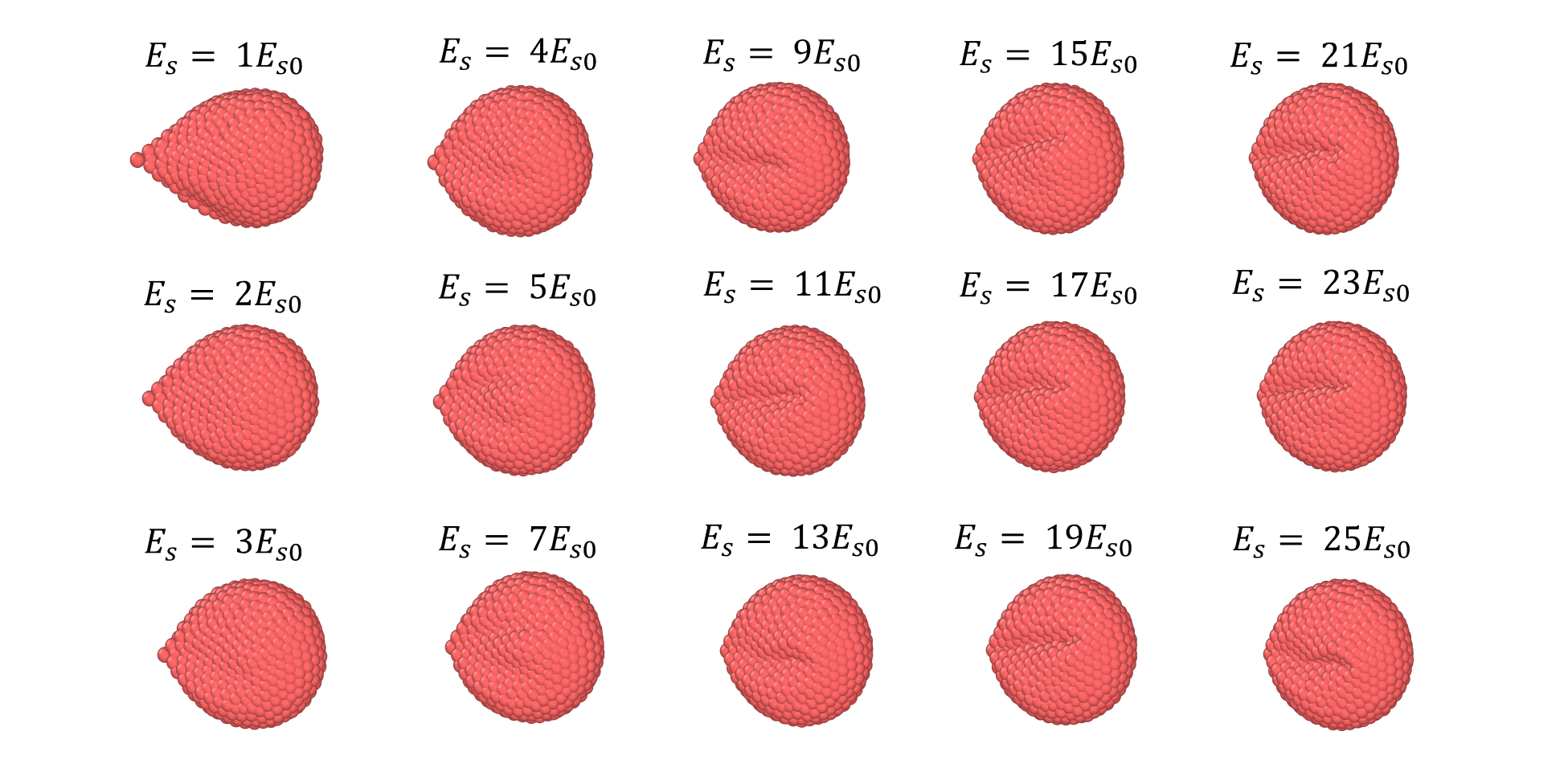

Supplement: S1 Fig — (TIF) [file pcbi.1012705.s006.tif]

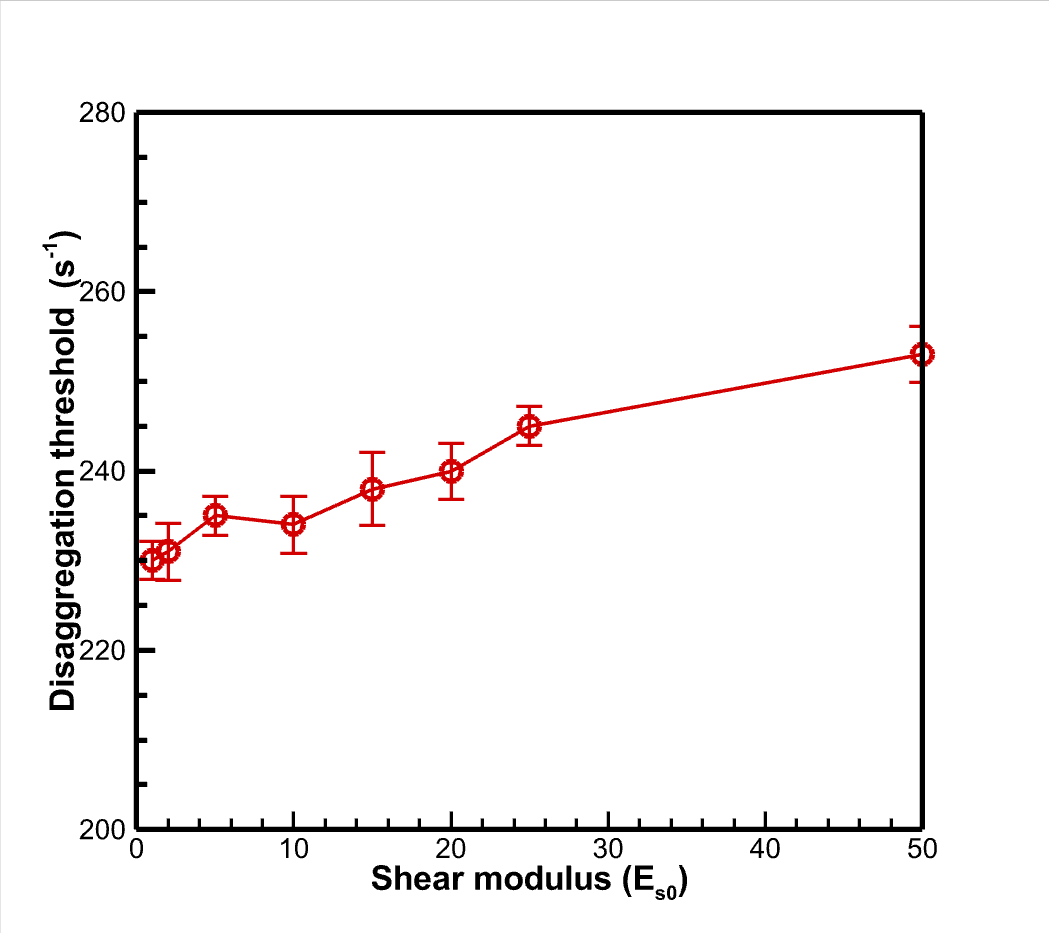

Supplement: S2 Fig — (TIF) [file pcbi.1012705.s007.tif]
